# The role of internal defects on anisotropic tensile failure of L-PBF AlSi10Mg alloys

**DOI:** 10.1038/s41598-023-39948-z

**Published:** 2023-09-06

**Authors:** Zhengkai Wu, Shengchuan Wu, Xi Gao, Ying Lin, Yanling Xue, Philip J. Withers

**Affiliations:** 1 State Key Laboratory of Rail Transit Vehicle System, Southwest Jiaotong University, Chengdu, 610031 China; 2 Shanghai Synchrotron Radiation Facility, Shanghai Advanced Research Institute, Chinese Academy of Sciences, Shanghai, 201204 China; 3 Henry Royce Institute, Department of Materials, The University of Manchester, Manchester, M13 9PL UK

**Keywords:** Metals and alloys, Mechanical properties, Imaging techniques, Characterization and analytical techniques

## Abstract

This paper investigates the effects of defects on tensile failure of additive manufactured AlSi10Mg alloy focusing particularly on the role of large pancake shaped loss of fusion (LOF) defects lying perpendicular to the build direction (BD). Time-lapse in situ synchrotron radiation X-ray micro-computed tomography during straining reveals how, when tested parallel to the BD, the LOF defects extend laterally with straining connecting to other defects and giving rise to low plasticity and an essentially brittle failure mode. When they are aligned edge-on to the straining direction, failure is characterised by a ductile cup-cone failure with significant elongation of the defects axially and extensive necking prior to failure. The soft fish-scale melt pool boundaries were also found to affect the fracture path. These results highlight the anisotropic effect of loss of fusion defects in controlling tensile ductility and the need to minimize their size and aspect ratio. In cases where these cannot be fully eliminated the component should be fabricated such that the BD is not aligned with the dominant in-service loading direction.

## Introduction

Due to its potential for high materials utilization, large design freedom, short lead-time and low energy consumption, additive manufacturing (AM) is attracting attention for structural design in the aerospace, automotive and railway sectors^1, 2^. However, as an emerging manufacturing technology, significant engineering challenges remain in the application of AM structures. These relate to scatter in performance due to such factors as residual stresses, heterogeneous microstructures, poor surface finish, and processing-induced defects. Most of these factors can be mitigated to some extent by the optimization of manufacturing parameters and subsequent heat treatments. Despite these efforts, the complex nature of the solidification process and the geometries thus formed mean that a certain level of residual defects are unavoidable^3, 4^. Recently, the formation of such porosity/defects and their effect on mechanical properties have received considerable attention^3–5^. Much of this has focused on the role of defects in significantly shortening the high cycle fatigue life of AM structures^6, 7^, while their effect on tensile fracture has received less consideration. Fadida et al.^8^ introduced artificial defects of different sizes into the centre of 4 mm diameter Ti–6Al–4V tensile samples made by laser powder bed fusion (L-PBF), finding that the ductility of samples with 600 μm or larger diameter pores is significantly reduced. Li et al.^9^ studied the influence of randomly generated defects on the tensile properties on selective laser melted 316L stainless steel, finding that upon increasing the scanning speed, the porosity of the material increases and the tensile strength decreases. Laursen et al.^10^ investigated the correlation between porosity and ductility, tensile strength, yield strength and elastic modulus for L-PBF AlSi10Mg, finding ductility to be most strongly affected.

The morphology of the entrained porosity tends to be characteristic of their formation mechanism^4, 11^. Of the three most common types of defects, i.e., gas-entrapped pores, keyhole and lack of fusion (LOF) defects, the ‘LOF defects’, being pancake shaped and relatively large in size, are considered to have the greatest impact on material properties^7, 12^. In this regard it should also be noted that the anisotropy in mechanical properties is one of the main characteristics of AM produced metals^13, 14^. In many studies, the volume of porosity is used to determine the quality and properties of AM products^15–19^, but this neglects the anisotropic three-dimensional (3D) nature of the defects. As a result, samples having the same volume porosity can show very different mechanical performance depending on their orientation to the build direction and load case^7, 20^. In this respect X-ray CT is invaluable as a means of quantifying the defect population, its distribution and anisotropy^21^. Further, in situ synchrotron radiation X-ray micro-computed tomography (SR-μCT) can image the growth and evolution of internal defects during deformation non-destructively, making it an excellent tool for studying the influence of 3D defects on the behaviour of AM metals^22–24^.

This study aims to elucidate the morphology of the largest defects as a function of build direction (BD) and to investigate their effect on the tensile properties for AM metals, looking in particular at AlSi10Mg alloy, one of the most commonly additively manufactured alloys. The behaviour of samples tested parallel and perpendicular to the BD is investigated to better understand how the anisotropic defects affect performance. To this end in situ synchrotron microtomography is employed to follow the growth of the entrained defects with straining. This work highlights that defects can largely be tolerated when they are extended parallel to the loading axis, but give rise to brittle behaviour when they lie perpendicular to the loading axis.

## Experimental methods

**Figure 1:**
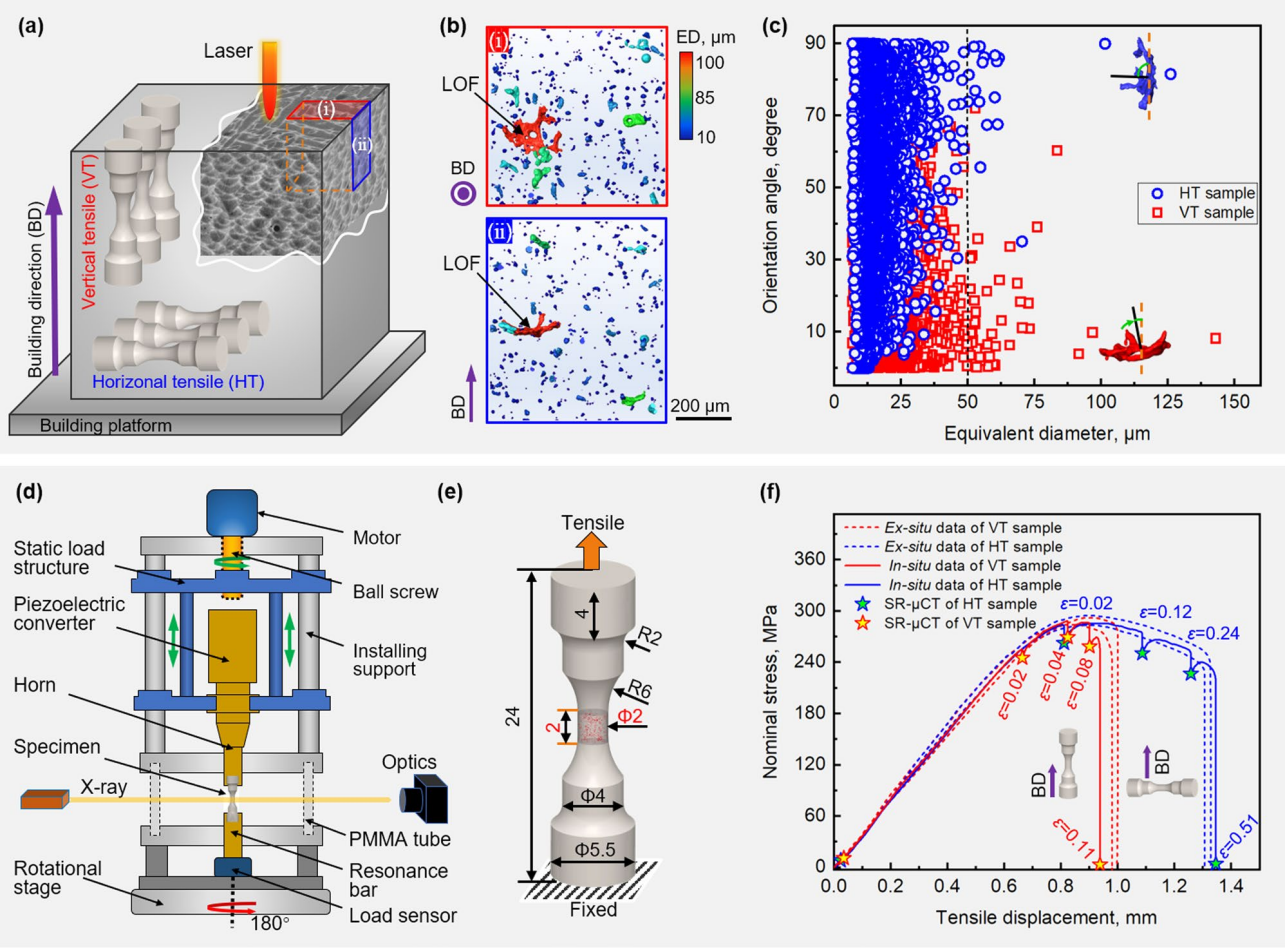
(**a**) Schematic showing the L-PBF block and the tensile samples extracted parallel (VT) and perpendicular (HT) to the BD. (**b**) volume renderings of the AM defects viewed along and perpendicular to the BD for a small region of interest. (**c**) Distribution of angle between the minor axis of the defect and the loading direction plotted against the equivalent diameter (ED, the diameter of a sphere having the same volume). (**d**) Schematic of the in situ testing rig installed at the SSRF. (**e**) Drawing of the tensile specimen in mm. (**f**) Stress-displacement curves showing the strains at which the in situ SR-μCT scans were acquired.

A 105 × 60 × 150 mm AlSi10Mg alloy block was produced on a BLT S310 (Xi’an Bright Laser Technologies Co., Ltd) L-PBF machine, which is equipped with a 500 W fibre laser with a beam quality M^2^ up to 1.1 and has a build area of 250 × 250 × 400 mm. The following parameters were adopted: laser power ~ 380 W, scanning speed ~ 1300 mm/s, hatch spacing ~ 150 μm and layer thickness ~ 50 μm, and a linear hatching strategy with a rotation of 67° between successive layers. Thermal stress relief (SR2, 300 °C/2 h/air cooling) was performed to relieve residual stresses according to the ASTM F3318 standard. This treatment, combined with the fact that upon cutting out the samples any remaining residual stresses are likely to be released means that the effect of residual stresses is likely to be small. As shown in Fig. 1a, vertical tensile (VT) and horizontal tensile (HT) specimens were extracted from the sample blocks by mechanical machining. By extracting the samples from a larger block and avoiding the corner and edge regions the aim was to achieve a fairly uniform distribution of defects both within a single sample, and from sample to sample. The in situ SR-μCT tests were carried out on a multifunctional loading rig (see Fig. 1d) installed on the BL13HB beamline station at the Shanghai Synchrotron Radiation Facility (SSRF). The in situ tensile specimens were designed to have a circular cross-section, of 2 mm diameter and 2 mm gauge length (see Fig. 1e). In order to determine the strain levels for 3D imaging, ex-situ tensile tests were performed beforehand to obtain the force–displacement curves of VT and HT samples. As shown in Fig. 1f, in order to follow the damage evolution with straining, SR-μCT imaging was performed at five deformation stages (denoted by star symbols) for both tests. At each step, μCT scans were acquired using a 26 keV photon energy beam, with an exposure time of 50 ms at pixel resolution of 3.25 μm. After the tensile tests, all the fractured samples were observed using a ZEISS SIGMA 500 scanning electron microscope (SEM) to identify the damage characteristics. In addition, samples parallel to the BD were cut from the as-stress relieved block and mechanically polished. Kroll’s etchant was used to characterize the microstructure by optical microscopy (OM) and SEM. In addition, cross-sectional samples were cut (parallel to the LD) from the post-mortem specimens to examine the micromechanical failure behavior. The microstructures were observed via electron backscatter diffraction (EBSD), using a JEOL-JSM-IT800 SEM equipped with a EBSD (Symmetry S2) system. The EBSD samples were mechanically polished using 0.05 µm silica suspension, followed by 2.5 h of Ar ion mill final polishing. The step size was 1 μm.

## Results and discussion

**Figure 2:**
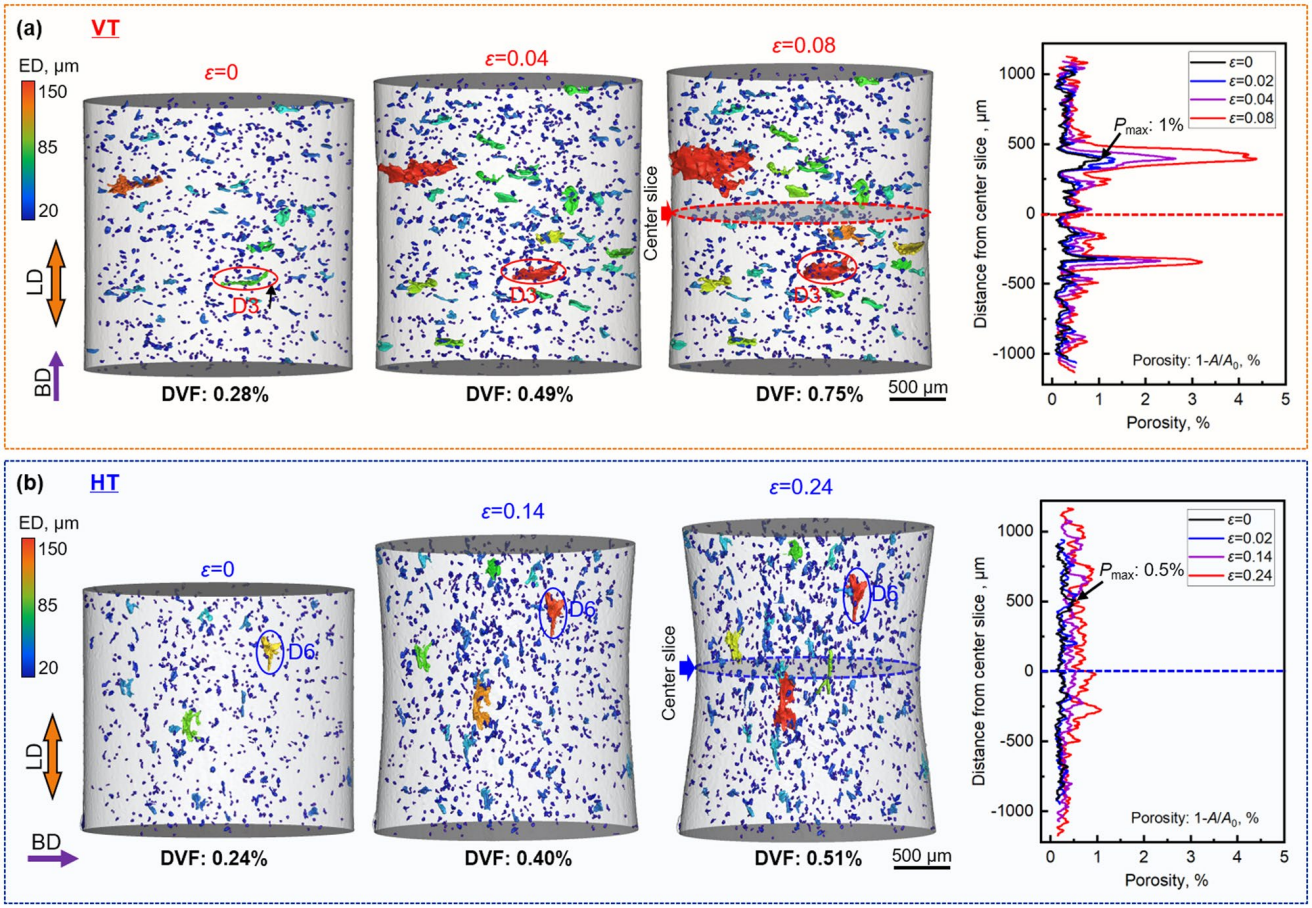
μCT volume renderings acquired during straining alongside plots of the cross-sectional porosity (P) as a function of distance from the centre for (**a**) VT and (**b**) HT samples. In the volume renderings the defects are coloured according to their equivalent diameter (ED).

It is evident from Fig. 1b that the projected area of LOF defects when viewed along the BD is much larger than when viewed perpendicular to the BD in common with previous work^25^. This is corroborated by the plot in Fig. 1c which shows the preferential alignment of these larger (> 50 μm) defects. Figure 1f indicates that, despite the orientation of the pancake shaped LOF defects, the tensile strength and stress–strain curve for the VT and HT specimens are similar to one another up until just before failure of the VT specimens at ~ 10% strain. However, it is evident that the ductility of the HT specimens is significantly higher than that of the VT specimens, which is consistent with previous studies^7, 26, 27^. The volume renderings presented in Fig. 2 clearly show the growth of the largest defects, along with the increase in overall defect volume fraction (DVF) as a function of the local true strain (denoted *ε* = ln(*A*_i_/*A*_c_) where *A*_i_ and *A*_c_ are the initial and current minimum cross-sectional areas) in the necked region. As one would expect, prior to straining the porosity of the two samples is similar (~ 0.25% vol.), but the orientation of the largest defects is clearly quite different. The distribution of defects was found to be fairly uniform within each sample and from sample to sample, as shown in Fig. 2 and in supplementary Fig. 1. After low level plastic straining, it is clear that the large flat defects in the VT sample have extended laterally to a much larger extent than those aligned with the LD in the HT sample. Further the overall damage fraction (DVF) is also higher despite the lower level of maximum strain. As a result, the pancake defects lying perpendicular to the LD lead to a sudden increase in the porosity cross-section (expressed as *P* = 1 − *A*_0_*/A,* where *A*_0_ and *A* are the cross-sectional area with defects and without defects). By contrast, the variations in the local porosity for the HT sample are much less extreme. The reduction in local cross-sectional area during tensile deformation for the VT sample, as well as the stress concentration arising from the crack like nature of these growing defects, lead to the rapid growth of defects perpendicular to the LD. This accelerates crack propagation and causes failure to occur for the VT sample at much lower strains, and with much less external necking, than for the HT sample.

**Figure 3:**
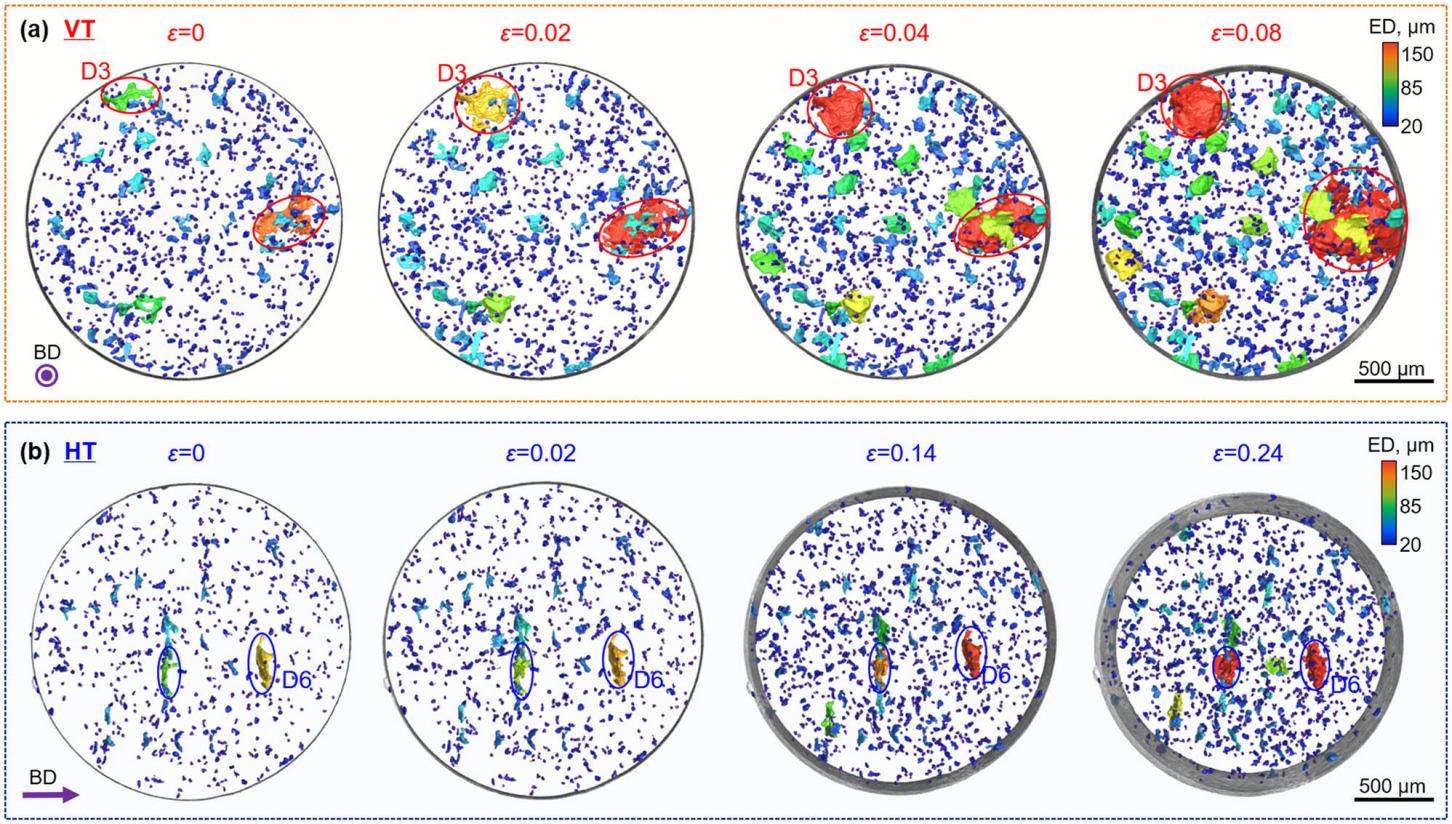
3D SR-μCT volume renderings viewed along LD showing defect evolution for (**a**) VT sample failed at *ε* = 0.11; (**b**) HT sample failed at *ε* = 0.51, where defects are coloured according to their ED.

It is evident from Fig. 3 that for the VT sample the pancake defects have extended laterally despite the low levels of plastic deformation, and that by 0.08 strain the largest defects have extended such that they become surface breaking, leading to brittle fracture at a strain of 0.11. It is noteworthy that the defects extend in a crack-like (lateral) manner rather than by ductile (axial) extension along the loading axis. By contrast, the HT sample exhibits extensive plastic deformation and necking with the projected area of the largest defects increasing only modestly, with little or no coalescence. Critically, the defects within the HT sample do not propagate sufficiently to become a surface crack, even when the necking strain has reached 24%.

**Figure 4:**
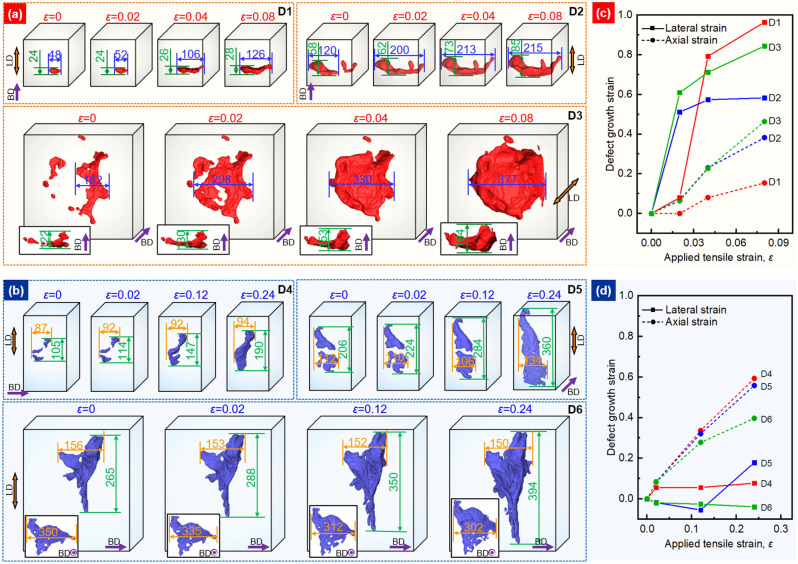
3D renderings of the defect evolution and coalescence as a function of longitudinal strain: (**a**) for defects D1, D2 and D3 within the VT sample; (**b**) for defects D4, D5 and D6 within the HT sample. The lateral and axial growth strains are shown as a function of applied tensile strain for (**c**) defects D1, D2, D3 in the VT sample and (**d**) defects D4, D5, D6 in the HT sample. All dimensions are in μm.

The manner of defect evolution in VT and HT samples is exemplified by the magnified views of six defects (D1–D6) as a function of loading strain, shown in Fig. 4a,b. In accordance with the conclusions drawn above, it is evident that the transverse extension of defects within the VT sample is much greater than for the HT sample. As the defects in the VT sample extend laterally, there is also evidence of defect coalescence phenomena, see for example D3, shown in Fig. 4a. By contrast, for defects in the HT sample, occasional defect coalescence of defects occurred mostly in the axial direction (such as for D4 and D5), while many defects elongated in the axial direction, without a significant change in shape (such as D6). Defect growth strains (denoted ln(*a*/*a*_0_)^28^, where *a* and *a*_0_ are the length or width of defects before and after deformation) in the axial and lateral directions can be used to analyze quantitatively the evolution of the defects^12^. Both lateral and axial growth strains of defect D1–D3 and D4–D6 are plotted as a function of applied tensile strain in Fig. 4c,d, respectively. It is evident that the transverse strain of the defect tends to be much higher than the axial strain for the VT sample, whereas the opposite is true for defects in the HT sample. For the VT samples with limited necking, the pancake defects grow laterally behaving more like brittle cracks than ductile voids. The axial stress accelerates the lateral expansion of these defects, leading to rapid crack propagation partly by connecting with small defects along its path. In contrast, the HT samples show essentially ductile failure, the maximum cross-section of the defect is always parallel to the LD, which is not conducive to the formation of cracks. Some elongated defects even contract slightly in the transverse direction (such as D6). This behaviour is an important factor leading to the high ductility of the HT samples.

**Figure 5:**
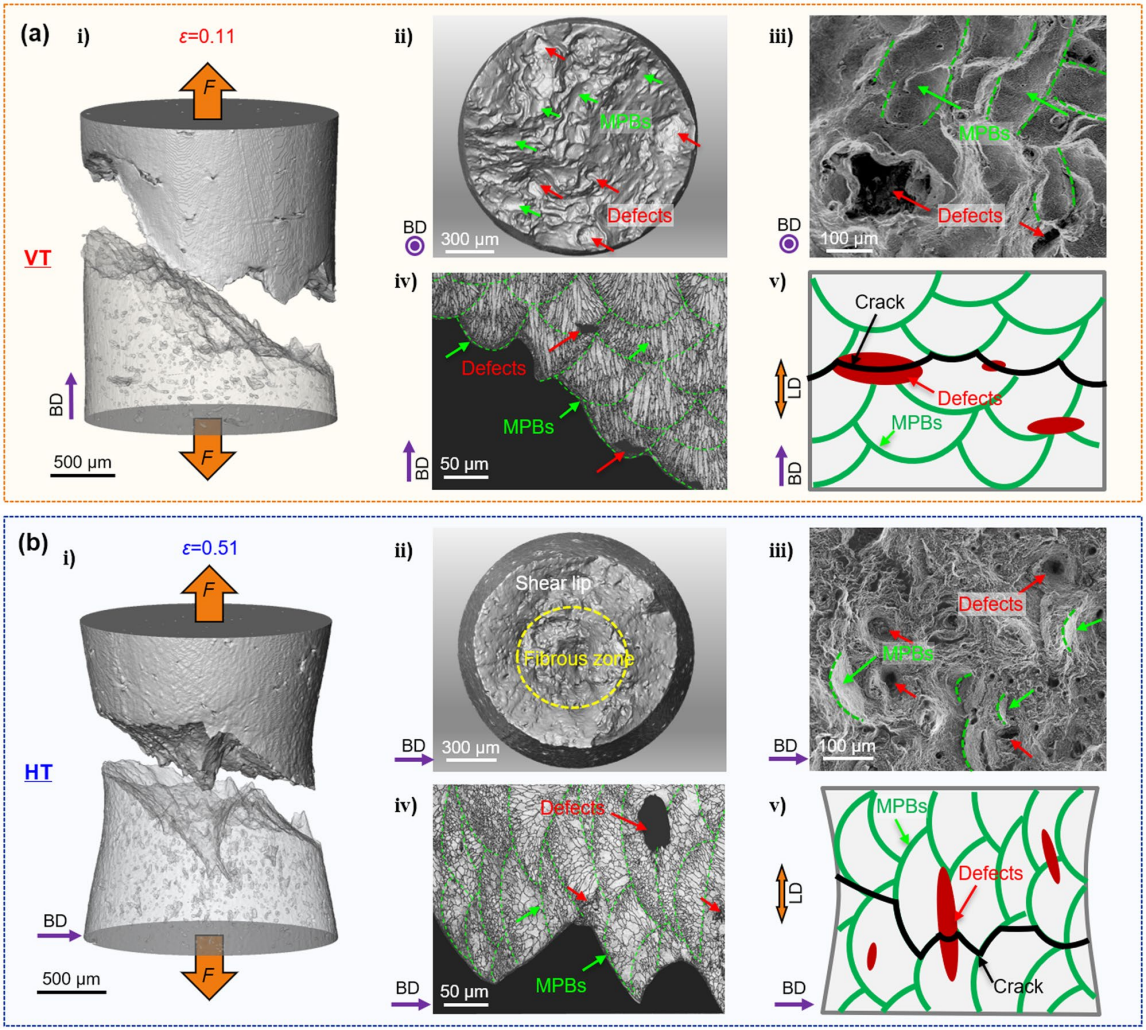
3D renderings of the fractured tensile sample: (**a**) VT and (**b**) HT samples showing (i) volume renderings of the failed samples (ii) 3D renderings of the fractured surfaces (iii) magnified SEM images of the fracture surfaces (iv) EBSD band contrast images for longitudinal sections just beneath the fracture surface and (v) Schematic representations of the respective tensile damage mechanisms.

Figure 5 further shows the final fracture surface morphologies complemented by videos from the X-ray projections recorded in situ (available in supplementary information). For the VT sample, these show characteristics of brittle rupture following defect evolution and crack propagation in the VT sample, whereas a more ductile failure with micro cracks initiating at the center following necking is observed for the HT sample. The fracture surface of the VT specimen is relatively smooth and contains large defects (see Fig. 5a ii), while that for the HT specimen exhibits a typical cup-cone fracture with shear lips (see Fig. 5b ii). The magnified region shown in Fig. 5a iii is consistent with failure from large defects for the VT sample, whereas the central (triaxial region) of the HT sample in Fig. 5b iii shows characteristic ductile void nucleation and growth. Finally, it should be pointed out that the influence of microstructure on the deformation and fracture behavior should not be ignored. The high cooling rate, strong temperature gradient and complex interaction between powder and molten pool during the layer-by-layer forming process gives rise to a complex hierarchically heterogeneous microstructure^29, 30^. In particular, the melt pool boundaries (MPB) are characterized by a coarser microstructure with a lower hardness (see supplementary Fig. 2). This can give rise to tearing along them leading to crack paths characteristic of the distribution of MPBs in the differently oriented samples^27, 31^. As shown in Fig. 5a, the MPBs (marked in green) when combined with the location of the defects (marked in red) gave rise to a relatively smooth crack growth path for the VT sample, which is conducive to rapid crack growth. By contrast, when the crack growth direction is parallel to the BD, the orientation of the MPBs and the edge-on defects, lead to a more tortuous crack growth path (see Fig. 5b). It can also be seen in supplementary Fig. 3 that the molten pool height of the HT sample is reduced, and the aspect ratio of the grains is significantly reduced leading to grain refinement. This indicates that the HT sample has undergone greater plastic deformation. As mentioned earlier, the stress relief treatment combined with the excision of small tensile samples mean that the effects of residual stresses in our case are likely to be small. However for real components residual stresses may combine with the stress concentrations caused by the LOF defects to further promote crack initiation and propagation^32, 33^, increasing the anisotropy of the mechanical properties.

## Conclusions

It is well known that entrained defects significantly limit the performance of metallic AM structures but that they cannot be completely eliminated during manufacture. In this paper, in situ SR-μCT has been used to quantitatively characterize the AM defect population for laser powder bed fusion (L-PBF) produced AlSi10Mg alloy, and their evolution tracked during tensile straining for samples taken from the same block (i.e. having the same average DVF) but oriented parallel (VT) and perpendicular (HT) to the build direction (BD). The main conclusions are:

1. While significantly reducing the ductility, the defects did not significantly affect the initial stress strain curves for the VT and HT samples because the reduction in area at peak strength was not sufficient to lower the strength significantly.
2. For the VT sample, the initial pancake shaped LOF defects were oriented perpendicular to the loading direction. Lateral crack-like propagation from the original defects was observed characteristic of a more brittle fracture process with limited plastic extension and necking due to early defect coalescence and propagation;
3. For the HT sample, the initial pancake shaped LOF defects were aligned nearly parallel to the LD. This showed essentially ductile tensile behaviour with significant plastic necking and axial growth of the defects.
4. In common with all AM produced material, the microstructure was found to be very heterogeneous showing fish scale patterns characteristic of the solidifying melt pool. The soft melt pool boundaries were found to affect the final crack path. The fish scale morphology combined with the location of the LOF defects gave rise to a more tortuous fracture surface for the HT sample compared to the VT sample.

In conclusion our findings complement earlier work that shows the critical role of the pancake-shaped loss of fusion defects in giving rise to anisotropic fatigue properties^25^. This work highlights their important role in limiting the tensile ductility, especially when loaded parallel to the build direction. This emphasizes the need to optimising the AM process to minimise their formation and control their orientation. Unlike fatigue resistance which can be improved by the careful use of compressive residual stresses introduced by peening for example, under tensile straining residual stresses tend to relax and so are less effective in mitigating the role of defects. However, recently, hipping^34^, magnetic field assistance^35^, laser shock peening^36^ and *in-situ* remelting^37^ during L-PBF process have been shown to be effective in reducing the defect level of AM parts, especially large-sized (50 µm effective diameter) defects. Furthermore, if these cannot be eliminated completely then it is necessary to consider the relationship between the build direction and the service loading scenario to minimise their influence on mechanical properties and to avoid brittle failure.

### Supplementary Information

Supplementary Figures.

Supplementary Video 1.

Supplementary Video 2.

## Data Availability

The datasets generated during and/or analysed during the current study are available from the corresponding author on reasonable request.
