# Supplementary material for: The role of internal defects on anisotropic tensile failure of L-PBF AlSi10Mg alloys
Source: Sci Rep. 2023 Sep 6;13:14681. doi: 10.1038/s41598-023-39948-z (PMC10482850; doi:10.1038/s41598-023-39948-z)
Supplement: Supplementary file 1 — Supplementary Figures. [file 41598_2023_39948_MOESM1_ESM.pdf]

Supplementary Information for

The role of internal defects on anisotropic tensile failure of L-PBF AlSi10Mg alloys

Zhengkai Wu [1], Shengchuan Wu [1, *], Xi Gao [1], Ying Lin [1], Yanling Xue [2], Philip J. Withers [3, *]

[1] *State Key Laboratory of Rail Transit Vehicle System, Southwest Jiaotong University, Chengdu 610031, China.*

[2] *Shanghai Synchrotron Radiation Facility, Shanghai Advanced Research Institute, Chinese Academy of Sciences, Shanghai 201204, China.*

[3] *Henry Royce Institute, Department of Materials, The University of Manchester, Manchester M13 9PL, United Kingdom.*

[*]Corresponding authors.

Email address: wusc@swjtu.edu.cn (S.C. Wu), p.j.withers@manchester.ac.uk (P.J. Withers)

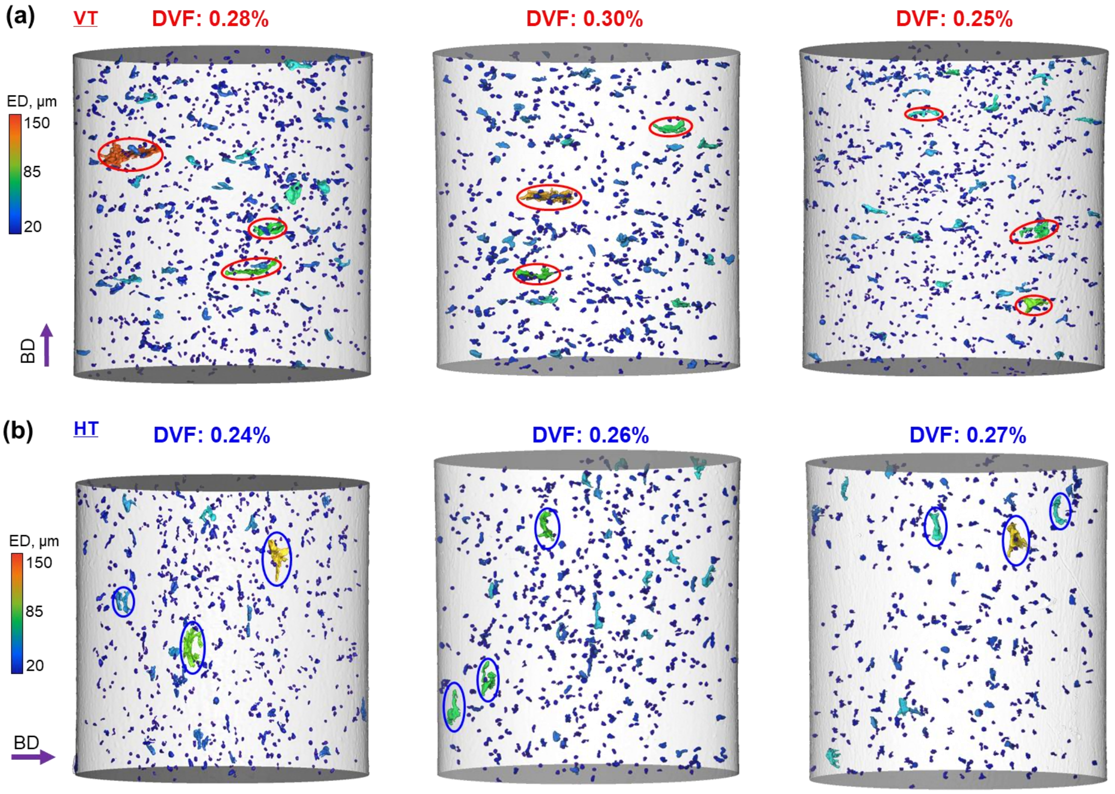

Supplementary Fig. 1. 3D SR-μCT volume renderings showing defect population for (a) VT samples; (b) HT samples, where defects are coloured by ED.

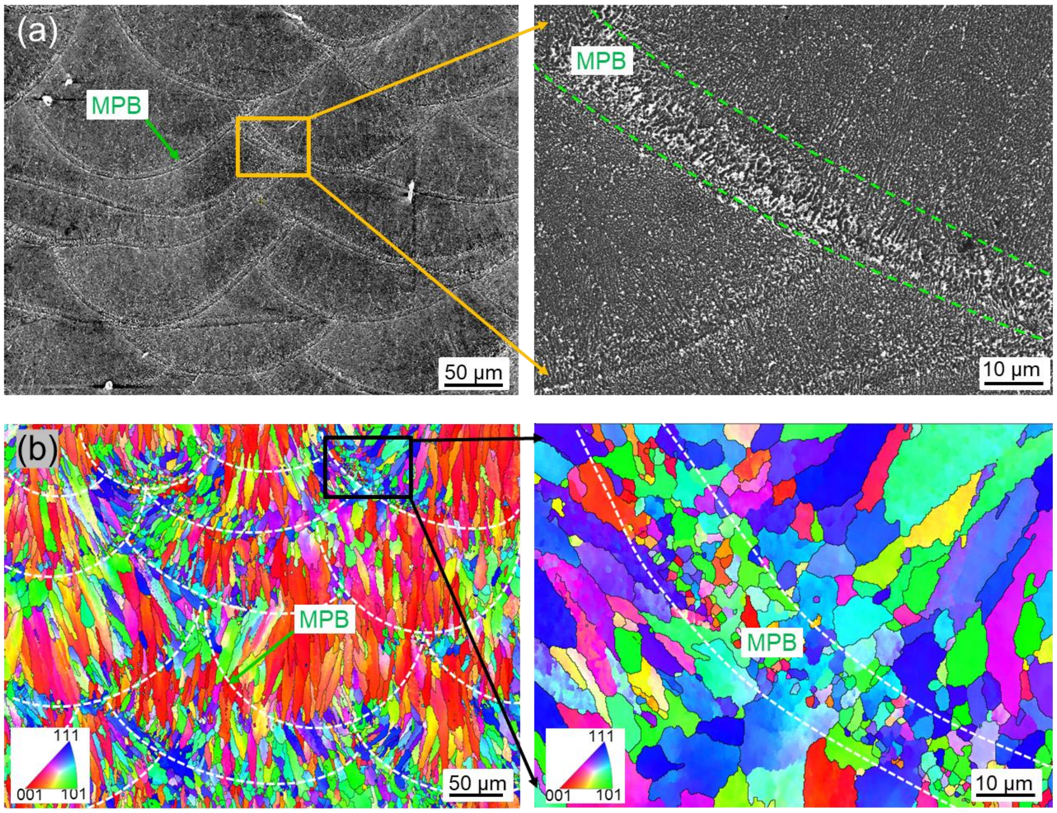

Supplementary Fig. 2. Typical microstructure of a L-PBF produced AlSi10Mg: (a) low and high magnification SEM images; (b) low and high magnification EBSD IPF maps.

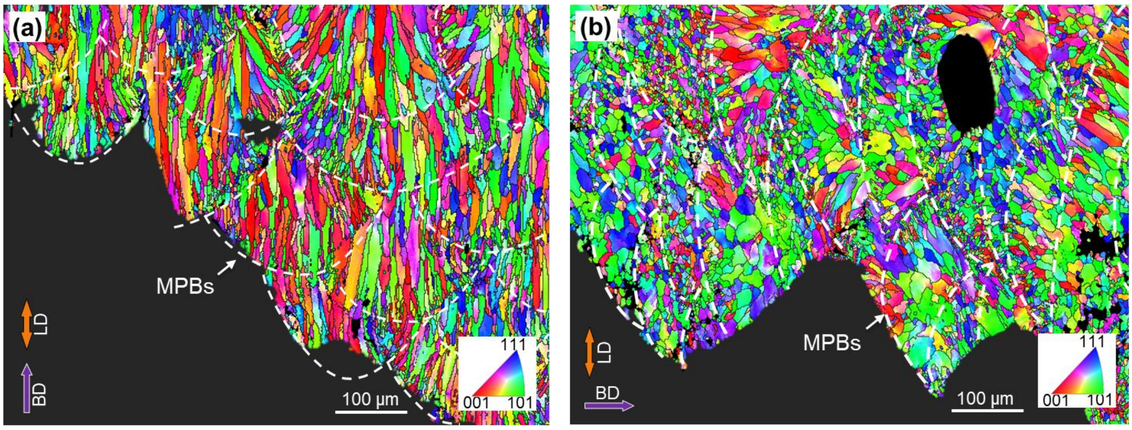

Supplementary Fig. 3: EBSD IPF maps for longitudinal sections just beneath the fracture surface: (a) VT sample; (b) HT sample
